# Supplementary material for: Growth and adaptation mechanisms of tumour spheroids with time-dependent oxygen availability
Source: PLoS Comput Biol. 2023 Jan 12;19(1):e1010833. doi: 10.1371/journal.pcbi.1010833 (PMC9876349; doi:10.1371/journal.pcbi.1010833)
Supplement: Attachment — Submitted filename: Murphy_Simpson_PCB_Sept2022.pdf [file pcbi.1010833.s005.pdf]

Title: Growth and adaptation mechanisms of tumour spheroids with time-dependent oxygen availability
Authors: Ryan Murphy and Mat Simpson
Ref: PCOMPBIOL-D-22-00961

Summary
This multidisciplinary study combines experimental and theoretical work (mathematical modelling and uncertainty quantification) to understand the impact of time-varying oxygen conditions on the growth dynamics of tumour spheroids. The experimental results are interesting, the models are novel and I am confident that the results will be of interest to the mathematical oncology community in particular but also to researchers developing modelling and computational approaches to study organoids. I particularly enjoyed the model built around the authors' Hypothesis 2. The paper is also extremely well written, easy to follow, with few typos, and figures that are beautifully clear. At the same time, I have a few relatively minor comments and suggestions that the authors might like to consider – these are listed below.

- Figure 1 (k,l): the schematics in these subplots show symmetry breaking of the spheroid. How robust are these findings? Were they observed in multiple cell lines? Also, to what extent can the Greenspan model (which assumes spherical symmetry) be used to describe their dynamics? What alternative modelling approaches might be better suited to these qualitative behaviours?
- The literature review in the introduction is rather short and could be expanded to review the different ways in which the degradation of dead or necrotic material is treated in mathematical models of tumour growth (see, for example, TD Lewin et al, Bull Math Biol (2018), which the authors cite but do not discuss in the introduction, and other articles by these authors).
- Equation (1) and hypothesis 2: what waste products might beta represent? How does the authors' treatment of beta differ from the ideas presented in Greenspan's original paper (they introduce a similar variable in their model, also denoted beta)?
- It would be helpful if the authors could clarify which bits of analysis of the Greenspan model (and its variant) are novel and which are reproduced from the work of other authors.
- Line 140: could the authors clarify what 'careful image processing' they did to demonstrate the good agreement between experimentally measured and predicted values of R_p(t)?
- What alternative mechanisms might explain the discrepancy between the estimates of p_i for spheroids grown in normoxia and hypoxia? Are the cell sizes the same in both conditions?
- I understand the rationale for using simple, time-dependent functional forms in equations (4.1)-(4.4) but have the authors considered alternative, more general functional forms that would enable predictions re spheroid response to other environmental conditions to be simulated? For example, might alpha depend on local levels of nutrient and waste metabolites? In this way, it might be possible to use studies on deoxygenation to predict (accurately) spheroid response's to re-oxygenation.

- The results in Figure 5(k) are intriguing and seem to resemble cell blebbing. Have the authors looked at the modelling literature re cell blebbing? See, for example, TE Woolley et al, Biomech Model Mechanobiol (2014). Additionally, might a multiphase model which distinguishes necrotic, live and fluid phases be appropriate here (see, for example, work by TD Lewin)?
- Given that the experimental data distinguishes different phases of the cell cycle, the authors may be interested in considering how fluctuating oxygen environments affect cell cycle progress. See, for example, GL Celora et al, J Theor Biol (2022).

In summary, I am happy to recommend the paper for publication, subject to minor revisions that address the minor comments and suggestions listed above.
