# Supplementary material for: Growth and adaptation mechanisms of tumour spheroids with time-dependent oxygen availability
Source: PLoS Comput Biol. 2023 Jan 12;19(1):e1010833. doi: 10.1371/journal.pcbi.1010833 (PMC9876349; doi:10.1371/journal.pcbi.1010833)
Supplement: Attachment — Submitted filename: Murphyetal2022_Cover_Letter_PLOSCB.pdf [file pcbi.1010833.s006.pdf]

Date October 17, 2022
Contact Ryan J. Murphy
Phone +61 435 892 555
E-mail r23.murphy@qut.edu.au
Subject Manuscript Revision: PCOMPBIOL-D-22-00961

Dr Ryan J. Murphy

School of Mathematical Sciences
Faculty of Science
Queensland University of Technology
GPO Box 2434, GP Campus
Brisbane, Queensland 4001 Australia

Professor Philip Maini
Academic Editor,
PLoS Computational Biology

Dear Professor Maini,

I am writing to submit a revised manuscript, “Growth and adaptation mechanisms of tumour spheroids with time-dependent oxygen availability”, that is being considered for publication in *PLoS Computational Biology*. We thank Dr James Osborne and the two anonymous referees for their valuable comments, and we appreciate the opportunity to address their comments.

We have revised the manuscript to incorporate all comments raised. To address the comments of Reviewer 1 we clarify that we interpret our results in the context of Greenspan’s mathematical model and that other interpretations are possible. To address the comments of Reviewer 2 we have added additional background to the literature review, clarified points raised, and extended the discussion on directions of future work. To address the comments of Dr James Osborne (Reviewer 3) we clarified the caption of Figure 2. All changes to the revised manuscript are highlighted in blue font and labelled in right margin of the text in agreement with the numbering in the brief discussion of the changes appended to this letter.

We hope that you find this revision suitable for publication in *PLoS Computational Biology*, and we look forward to hearing from you.

Yours sincerely,

R. Murphy

Dr Ryan J. Murphy

Reviewer 1

This is a very interesting study about an area of special importance to researchers who utilise spheroids for research. Overall, I think this is solidly put together and has interesting findings, but I worry slightly about over-interpretation of borderline results, and would like some of these aspects clarified. In no particular order, I have the following suggestions and queries:

Response: We thank Reviewer 1 for their positive comments and for the opportunity to address their queries.

(R1.1) As a general guiding principle, I am a strong believer that Occam's razor should always be observed when constructing mathematical models and entities should not be multiplied without necessity. The reason spheroid models have been relatively simply to date is because, as the authors observe, we are typically dealing with snapshots and our existing models describe these well in terms of oxygen-limited diffusion. What the authors propose in interesting (and indeed, even likely) but I would caution tempering some of the claims or providing more evidence for them.

Response: We are also strong believers in Occam's razor when constructing mathematical models. This modelling philosophy is a key reason for analysing our experiments starting with the seminal and relatively simple Greenspan mathematical model (R1.1a, Page 4 Lines 91–92). We agree that spheroid studies typically deal with snapshots and that many results are described well in terms of oxygen-limited diffusion. For example, Grimes et al. 2013 and Gomes et al. 2016 estimate the oxygen partial pressure within individual spheroid snapshots at specific points in time. However, in this study we make an important distinction by explicitly considering the temporal evolution of overall spheroid size and structure at multiple time points. It is this distinction, in the context of Greenspan's mathematical model, which gives rise to our claims and results (R1.1b Page 4 Lines 74–87, R1.1c Page 17 Lines 358–359). We discuss this in greater detail in the below points.

(R1.2) The authors state "In phase (ii) cells in the central region of the spheroid arrest in G1 phase while cells at the periphery continue to proliferate resulting in inhibited growth (Figure 1b). This arrested region is thought to arise due to spatial differences in nutrient availability, possibly oxygen, and/or a build up of metabolic waste from cells. In phase (iii) the spheroid is characterised by three regions: a central region composed of a necrotic core, $0 < r < R_n(t)$; an intermediate region of living but proliferation-inhibited cells" - this seems somewhat contradictory to what is typically observed in both spheroids and in situ tumours. One of the distinguishing features of tumour cells is their extreme hardiness and resistance to quiescence (10.1016/j.cell.2011.02.013 and many other publications). In terms of oxygen, cancer cells continue replication unimpeded up to extremely low oxygen pressure of around 0.5mmHg before mitotic arrest (10.1093/jnci/93.4.266), which was also seen with spheroids in references the authors cite (10.1371/journal.pone.0153692) and has also been seen in situ cancers, including glioblastoma (10.1038/s41416-020-1021-5): while a proliferation impeded region does exist for non-necrotic cells in spheroids at oxygen pressures below the necrotic region, it has a very small radial extent. My suspicion is that the authors may not be considering the image analysis of their fluorescence data and fully de-convoluting it to avoid bleed-through; this would involve analysing expression across all channels and subtracting a noise-gated version. MATLAB allows this pretty easily, but the figure in 1c looks like a tumour ellipsoid which has an entirely different oxygen profile than a spheroid (10.1098/rsif.2018.0256) so I'd like the authors to address these aspects if they could.

Response: We address this comment in three parts. First, we note that we are considering spheroids that are composed of melanoma cell lines. We do not believe that comparing melanoma spheroids to *in situ* tumours such as glioblastoma, and even spheroids formed by other cell types, is warranted. Each cell line may respond differently to oxygen, nutrients and waste. Furthermore, *in situ* tumours involved multiple cell types with other processes (e.g. blood flow and immune response) that are completely absent in a melanoma cell line spheroid. Interesting future work would be to explore other cell lines and *in situ* tumours (R1.2a, Page 18 Lines 403–406).

Second, it is common to experimentally characterise melanoma spheroids by three phases of growth resulting in a spheroid with three regions (necrotic core, inhibited region, and proliferative region). Using fluorescence ubiquitination based cell cycle indicator (FUCCI) transduced melanoma cells to form spheroids allows us to observe the cell cycle distribution within each spheroid. Then using confocal microscopy and image processing on MATLAB we identify outer, inhibited, necrotic, and hypoxic regions. Experimental images with the boundaries of the necrotic, hypoxic, and inhibited region overlayed are presented in Supplementary Discussion A.2 (R1.2b, Supplementary Page 4 Lines 48–59). These results are supported by our previous studies clearly demonstrating the existence of three regions (Haass et al. 2014, Spoerri et al. 2017, Murphy et al. 2022, Browning et al. 2022) (R1.2c, Page 2 Lines 54–55, R1.2g, Page 2 Lines 60–63). Furthermore, results presented here from optical sectioning of spheroids using confocal microscopy are consistent with results obtained from confocal imaging of spheroid cryosections (Gunasingh et al. JOVE 2022) (R1.2d, Page 23 Lines 542–543). We clarify that we identify the boundary between the inhibited and proliferating region to be the radial position at which 20% of cells are cycling (Browning et al. Zenodo 2021) (R1.2e, Page 24 Lines 572–574). However, even with this clarification there are annular regions within spheroids for which all cells appear arrested. We also note that Greenspan's seminal mathematical model developed to model a generic spheroid explicitly incorporates the three phases that we find (R1.2c, Page 2 Lines 54–55).

Third, the term spheroid refers to an object that is not perfectly spherical. Therefore, we do not expect our tumour spheroids to be perfectly spherically symmetric. It is common in mathematical modelling of tumour spheroids to make a simplifying assumption of spherical symmetry. We restrict our use of Greenspan's mathematical model, and variants thereof, to situations where it appears reasonable to assume spherical symmetry in the first instance (R1.4a Page 16 Lines 322–324, R1.4d Page 18 Lines 390–391). Finally, we now emphasise in the discussion that our results depend on the assumption of spherical symmetry and that interesting future work would be to explore the impact of relaxing this assumption (R1.2f, Page 18 Lines 401–403).

(R1.3) Part of the problem might be the use of pimonidazole staining as a proxy for hypoxia - although it has long been used, there are problems in interpreting it (10.1667/RR1305.1, 10.1016/s0167-8140(03)00010-0) as its binding properties fluctuate greatly with pO2, unlike EF5 which remains constant. Have the authors taken stock of this? If its possible to replicate the experiments, I'd suggest doing so with EF5 because of the problems Koch describes with the staining agent itself; if it varies with pO2, then experiments on hypoxia with it might be badly confounded.

Response: Throughout this study we use pimonidazole staining consistently across all conditions (R1.3a, Page 23 Lines 545–551). This includes using the same experimental procedure and confocal microscopy imaging settings. We have not repeated the analysis using EF5 as we do not believe that our results are confounded due to the following. Hypoxia measurements are only used quantitatively in this study to validate estimates of

the oxygen partial pressure within the spheroid. Specifically, we compare the measured hypoxic radius and the predicted hypoxic radius that is calculated using measurements of the outer radius, $R_{\mathrm{o}}(t)$, and necrotic radius, $R_{\mathrm{n}}(t)$ (Figure 3e). These results suggest that the estimated oxygen partial pressure within each spheroid are reasonable and that the pimonidazole staining and image processing is appropriate for our purposes (R1.3b, Page 9 Lines 166–169). In the remainder of the study pimonidazole is primarily used as a visual aid in experimental images (R1.3c, Page 14 Lines 278–280). Measurements of pimonidazole staining are not used to estimate parameter of Greenspan's mathematical model. We agree that interesting future work would be to repeat the experiments using EF5 staining and compare results (R1.2a, Page 18 Lines 403–406).

(R1.4) The necrotic core "exiting" the spheroid seems rather interesting. The video appears to be bright-field microscopy, and I have an idea of what might be happening: in the original work of Sutherland and Fryer etc, they demonstrated that spheroids eventually split apart. The necrotic cells within the core lyse, and perturbations in the suspending fluid mean unbalanced pressures occur, with the spheroid "bursting". I believe that is what the investigators are seeing, but at this point the clump of cells can not longer be said to be a spheroid. From the methods section, I don't seem to think the spheroids were made in a spinner culture or similar (10.1517/14712598.2012.707181) so it is unlikely they will maintain their approximately spherical state. The non-bursting video seems to show the spheroid becoming an ellipsoid (10.1098/rsif.2018.0256) so the figures showing no morphogenic changes from a spherical shape in figure 1 are misleading; if the shape of the spheroid departs from approximately spherical symmetry due to unbalanced pressures or irregularities in growth media, it will very quickly depart from the assumptions of perfect diffusion. The bubbles on the bright field microscopy indicate some media disturbances too.

Response: The necrotic core exiting is indeed interesting. We note that this behaviour only occurs under certain conditions. Spheroids in this study retain their approximately spherical shape under normoxic conditions, hypoxic conditions, and de-oxygenation experiments. Spheroids also retain their approximately spherical shape in re-oxygenation experiments for the WM793b cell line. Supplementary movies, captured using brightfield microscopy (Supplementary Discussion H: Supplementary Movie Descriptions) are included to represent WM983b re-oxygenation experiments where there is movement of necrotic material. We do not analyse such experiments using a mathematical model. We restrict our use of Greenspan's mathematical model to situations where it appears reasonable to assume spherical symmetry in the first instance (R1.4a Page 16 Lines 322–324, R1.4d Page 18 Lines 390–391). Further for WM164 re-oxygenation experiments, where it appears reasonable to assume spherical symmetry in the first instance (Figure S16, S17), we interpret results using Greenspan's model but note that additional care should be exercised as brightfield timelapse images show that mass from the necrotic core can move to the periphery (R1.4b, Page 16 Lines 329–332, R1.4c Page 46 Lines 379–381). To make Figure 1 clearer we update the caption and add label to highlight that schematics represent WM983b re-oxygenation experiments with necrotic core movement. Furthermore, in the caption of Figure 1 we now refer the reader to Supplementary Material A.2 to view supplementary confocal and brightfield images which have lines representing the identified proliferating, inhibited and hypoxic regions overlayed (R1.2b, Supplementary Page 4 Lines 48–59).

(R1.5) I do not think, based on the considerations of the above points, that the authors have proven "Oxygen diffusion alone is insufficient to describe spheroid growth" - if anything, all of the phenomena they describe in this work are consistent with pO2 limited diffusion.

It would be interesting to find other factors (which presumably must exist) but as it stands I do not think the authors have overcome the threshold of proof to demonstrate this.

Response: We now place our results, including the statement that oxygen diffusion alone is insufficient to describe spheroid growth, in the context of interpreting the experimental data using Greenspan's mathematical model (R1.5a Page 5 Lines 122–123, R1.2f Page 18 Lines 401–403). We believe that a reasonable interpretation of Greenspan's mathematical model is that p_i should be constant and independent of the external oxygen partial pressure (Page 5 Lines 140–143). As estimates of p_i are not consistent with this interpretation we suggest that other mechanisms, possibly metabolic waste production and diffusion, are more likely to drive the formation and growth of the inhibited region. Alternatively, if one was to restrict themselves to the notion that oxygen must be directly responsible for formation and growth of the inhibited region it is unclear why cells within spheroids grown in normoxia arrest at a higher oxygen partial pressure than cells within spheroids grown in hypoxia. It seems to be more plausible that an alternative mechanism is relevant (Page 9 Lines 176–185).

(R1.6) Greenspan's model, it must be noted, is a very old but useful general model for spherical oxygen diffusion, but it has long since been superseded. However, following on from point 4, the primary assumption that "The model assumes each spheroid is spherically symmetric and maintained by cell-cell adhesion or surface tension" is quite clearly contradicted by the evidence the authors have provided; in this case, all spherically symmetric calculations rapidly break down, but that would be predicted by the nature of oxygen diffusion itself and basically amounts to a solution of poisson's equation in a unique geometry.

Response: We agree that Greenspan‘s model is a useful general model to describe spheroid formation. However, we do not agree that Greenspan's model has long since been superseded. Consistent with Reviewer 1's guiding principle of Occam's razor, we choose to start with Greenspan's mathematical model as it is simple, describes the three phases of growth in normoxia and hypoxia experiments, and has five parameters each of which are physically and biologically interpretable (R1.1a Page 4 Lines 91–92, R2.2a Page 4 Lines 92–95). Surprisingly, while the model has been extended many times theoretically it is only in our recent study that, to the best of our knowledge, Greenspan's mathematical model was quantitatively connected to data (Murphy et al. 2022). Further in Murphy et al. 2022 we show that parameters can be estimated using the data collected in this study. Other more complicated spheroids models which have followed Greenspan's model face challenges of parameter identifiability with data that we have collected in this study (Browning et al. 2022, Jin et al. 2022) (R2.2a Page 4 Lines 92–95).

With regards to following on from point 4, we emphasise that we do not mathematically model the necrotic core exiting. We restrict our use of Greenspan's mathematical model, and variants thereof, to situations where is appears reasonable to assume spherical symmetry in the first instance (R1.4a Page 16 Lines 322–324, R1.4d Page 18 Lines 390–391).

(R1.7) As I understand it, the results shown in Figure 2 are consistent for the projected and measured growth dynamics in multiple cell lines shown in (doi.org/10.1371/journal.pone.0153692) - using this model or similar, you would expect these growth dynamics, but the lack of radial measures in the figure makes it difficult to directly compare and should be labelled. The general growth dynamics of spheroids are well understood including their phases (Cancer Res 1983, 43(2): 556–560, doi not readily available) and in all cases, a

sigmoid function is typically obtained (10.1111/j.1365-2184.1994.tb01407.x) - however, from what I can observe, none of the spheroids were grown to plateau stage, which unfortunately reduces ones ability to make strong generalisations from the presented data. Spheroids in this work seem very young and small, and it is difficult to see differences in properties until they grow considerably, as indeed their quasi-linear / plateau point varies by OCR and pO2 at spheroid edge.

Response: In the caption of Figure 2 we now refer the reader to Figure 1e to view a schematic of describing radial measurements, including the outer, inhibited, hypoxic and necrotic radius. Furthermore, in the caption of Figure 2 we refer the reader to Supplementary Material A.2 to view confocal and brightfield images which have lines representing the identified proliferating, inhibited and hypoxic regions overlayed (R1.2b, Supplementary Page 4 Lines 48–59). All measurements are made freely available on a GitHub repository listed in the manuscript should one wish to explore examine this dataset with different models, such as the model in the reference.

We agree that the general growth dynamics of spheroid's overall sizes are well understood, and that if spheroid's are grown sufficiently long they are typically well-represented by sigmoidal functions. This is indeed the case for melanoma spheroids generated by the cell lines considered in this study. Specifically, by fitting Greenspan's mathematical model to experimental data collected under normoxic and hypoxic conditions we observe that spheroid's are close to or already at their long-time size and structure (Figure 3i-o, S24). We note that the experimental duration in this study was informed by our previous study where we explore different experimental designs to understand when Greenspan's model parameters are practically identifiable and can be estimated (Murphy et al. 2022) (R2.2a Page 4 Lines 92–95, R1.7a Page 23 Lines 529–532).

(R1.8) Figures 3 and 4 and very difficult to follow, and the caption barely helps. If you are cross comparing two hypotheses, it might be easier to include a table in your graphic explicitly stated differences.

Response: We have now rephrased and added additional explanation to the captions of Figures 3 and 4. Further, we have updated the schematic in Figure 3b so that it is clearer that R_{i} is implictly defined via a waste threshold β_{i}. As captions are already long, Reviewer 2 refers to the figures as beautifully clear, and both Reviewer 2 and 3 both found the study to be well written and easy to follow, we have not introduced an additional table.

(R1.9) Again, the adaptations to hypoxia is in my opinion more readily explained by simple non-sphericity than by esoteric mechanisms. I have no doubt that processes like waste disposal play some role, but on the basis of the evidence presented, I do not feel the authors have even considered the huge role a departure from sphericity has on re-oxygenation and even the physical stability of spheroids.

Response: We restrict our use of Greenspan's mathematical model, and variants thereof, to situations where it appears reasonable to assume spherical symmetry in the first instance. This includes adaptations to hypoxia in deoxygenation experiments where we observe that spheroids remain approximately spherically symmetric. We do not mathematically model re-oxygenation experiments where the necrotic core moves (R1.4a Page 16 Lines 322–324, R1.4d Page 18 Lines 390–391).

In our study we first analyse spheroids grown in normoxic and hypoxic conditions. Our analysis suggests that oxygen diffusion alone is not sufficient to describe spheroid growth, at least in the context of Greenspan's mathematical model (see response to point R1.5). Therefore, it seems most appropriate to build on the model which incorporates oxygen

and waste mechanisms (hypothesis 2) when constructing the deoxygenation and re-oxygenation mathematical models (Page 10 Lines 216–217).

(R1.10) While I commend the authors for their work so far, I think these objections would have to be either addressed or conceded before this work can be published. Occam's razor should not be forgotten, and explanations requiring the least number of additional assumptions should be favoured, at least unless there is solid evidence that this is not sustainable. In this case, I am not yet convinced by the evidence presented. Oxygen diffusion historically explains the dynamics of avascular tumours and spheroids to the levels we have been able to measure with probes and stains. Due to experimental limitations, this is generally the level to which we can resolve. The authors assertions that diffusion is insufficient to explain what they observed I believe is less likely than the competing idea that their model is overly reliant on spherical symmetry compounded with difficultly interpreting confocal staining. I am of course willing to see revisions and again congratulate the authors on an ambitious undertaking.

Response: We thank Reviewer 1 for their detailed review. In addressing the above points we believe that we have demonstrated that oxygen dynamics alone are not sufficient to describe the observations in this study in the context of Greenspan's mathematical model. By placing our results in the context of Greenspan's model we have aimed to suitably caveat our results for further explorations relaxing assumptions of spherical symmetry (Page 18 Lines 401–403).

Reviewer 2

Summary: This multidisciplinary study combines experimental and theoretical work (mathematical modelling and uncertainty quantification) to understand the impact of time-varying oxygen conditions on the growth dynamics of tumour spheroids. The experimental results are interesting, the models are novel and I am confident that the results will be of interest to the mathematical oncology community in particular but also to researchers developing modelling and computational approaches to study organoids. I particularly enjoyed the model built around the authors' Hypothesis 2. The paper is also extremely well written, easy to follow, with few typos, and figures that are beautifully clear. At the same time, I have a few relatively minor comments and suggestions that the authors might like to consider – these are listed below.

Response: We thank Reviewer 2 for their positive summary of the paper.

(R2.1) Figure 1 (k,l): the schematics in these subplots show symmetry breaking of the spheroid. How robust are these findings? Were they observed in multiple cell lines?Also, to what extent can the Greenspan model (which assumes spherical symmetry) be used to describe their dynamics? What alternative modelling approaches might be better suited to these qualitative behaviours?

Response: The results from re-oxygenation experiments represented in the schematic of Figure 1 (k,l) describing symmetry breaking of the spheroid are reproducible under certain conditions. Figure 1k corresponds to results from WM983b re-oxygenation experiments where the oxygen partial pressure is increased from 2% to 21% oxygen 2.5 days after seeding (Figure 1k, 5k, Movie S1). Similar behaviour is observed using WM983b spheroids when re-oxygenation occurs 5.5 days after seeding, albeit the necrotic core does not exit as a whole object (Figure 1l, 5m, Movie S2). In WM164 spheroids mass from the necrotic core appears to move from the necrotic core to the periphery and exit (Movie S3). This movement was not observed for the WM793b cell line. It appears to be an open question how robust these finding are to other cell lines and conditions (Page 18 Lines 387–388). In the manuscript we now update the caption of Figure 1 to make clear that the schematics in Figure 1k,l occur under certain conditions. Further, we add a label in Figure 1 to highlight that schematics correspond to re-oxygenation experiments where spherical symmetry is lost due to necrotic core movement.

In experiments where we observe symmetry breaking we do not use Greenspan's mathematical model (R1.4a Page 16 Lines 322–324, R1.4d Page 18 Lines 390–391). It is an open question how to model such symmetry breaking phenomena. In the discussion we now outline that interesting future work is to ascertain the physical and biological mechanisms that could explain these observations. Interesting future work is to explore alternative modelling approaches, for example multiphase model's, studies on cellular blebbing, or compound droplet models (R2.1a Page 18 Lines 390–394).

(R2.2) The literature review in the introduction is rather short and could be expanded to review the different ways in which the degradation of dead or necrotic material is treated in mathematical models of tumour growth (see, for example, TD Lewin et al, Bull Math Biol (2018), which the authors cite but do not discuss in the introduction, and other articles by these authors).

Response: We have now expanded the literature review in the introduction, including the work of Lewin et al. 2018 (Page 4 Lines 74–87). Further, we have added that interesting future work would be to explore models with different treatments of necrotic material (Page 18 Lines 390–394) .

(R2.3) Equation (1) and hypothesis 2: what waste products might beta represent? How does the authors' treatment of beta differ from the ideas presented in Greenspan's original paper (they introduce a similar variable in their model, also denoted beta)?

Response: In our study we treat waste products identical to that introduced in Greenspan's original paper (Supplementary Page 28 Lines 140–141). Hence, we also use β to denote waste products. We assume that β represents diffusible metabolic waste that is produced by living cells (Page 8 Line 144).

(R2.4) It would be helpful if the authors could clarify which bits of analysis of the Greenspan model (and its variant) are novel and which are reproduced from the work of other authors.

Response: We have now clarified which parts of the analysis of Greenspan's model and its variants are novel and which parts are reproduced from the work of other authors. Firstly, the deoxygenation and re-oxygenation mathematical models are both novel. While comparisons can be made to the extension of Greenspan's model presented in Lewin et al. 2018 the mechanisms and results are significantly different. In Lewin et al. 2018 the oxygen partial pressure within the spheroid changes due to an instantaneous loss of living cells from radiation induced cell death. In our study, the oxygen partial pressure within the spheroid changes because the external oxygen partial pressure changes with no instantaneous loss of living cells. Furthermore, the study of Lewin et al. 2018 is a theoretical study. In contrast, here we develop new mathematical models and connect them to novel experimental data that we collected (Page 4 Lines 97–101) .

Results in Figure 2c-e estimating the outer radius when the necrotic region forms, R_{c}, the oxygen consumption rate, α, and validating the oxygen profile by comparing the measured and predicted hypoxic radii R_{p} follow the methodology of Grimes et al. 2013. Results estimating the oxygen partial pressure in Figure 2f-h are analogous to Gomes et al. 2016. However, subsequent analysis exploring the temporal evolution of the overall size and structure of spheroid's in different oxygen conditions using Greenspan's mathematical model (and deoxygenation and re-oxygenation variants) is novel (Page 4 Lines 74–87) .

(R2.5) Line 140: could the authors clarify what 'careful image processing' they did to demonstrate the good agreement between experimentally measured and predicted values of $R_{\mathrm{p}}(t)$?

Response: We now clarify that image processing to measure $R_{\mathrm{p}}(t)$ was challenging due to gradients in the pimonidazole signal. We refer the reader to Supplementary Discussion Section B (Page 9 Lines 164–166), where we detail additional image processing steps to account for the gradient in the pimonidazole staining (Supplementary Page 24 Lines 77–86).

(R2.6) What alternative mechanisms might explain the discrepancy between the estimates of p_{i} for spheroids grown in normoxia and hypoxia? Are the cell sizes the same in both conditions?

Response: We believe that a reasonable interpretation of Greenspan's mathematical model is that p_{i} should be constant and independent of the external oxygen partial pressure (R1.5b Page 5 Lines 140–143). As estimates of p_{i} are not consistent with this interpretation we suggest that other mechanisms, possibly metabolic waste production and diffusion, could be a more reasonable mechanism to describe the formation and

growth of the inhibited region. Alternatively, if one was to restrict themselves to the notion that oxygen must be directly responsible for formation and growth of the inhibited region it is unclear why cells within spheroids grown in normoxia arrest at a higher oxygen partial pressure than cells within spheroids grown in hypoxia. It seems to be more plausible that an alternative mechanism is relevant (Page 9 Lines 176–185). Any differences in cell size are expected to be captured in estimates of the oxygen consumption rate per unit tumour mass, α (R2.6a Supplementary Page 26 Lines 106–108). It would be interesting to explore other mechanisms that might explain this discrepancy (R1.2a Page 18 Lines 403–406).

(R2.7) I understand the rationale for using simple, time-dependent functional forms in equations (4.1)-(4.4) but have the authors considered alternative, more general functional forms that would enable predictions re spheroid response to other environmental conditions to be simulated? For example, might alpha depend on local levels of nutrient and waste metabolites? In this way, it might be possible to use studies on deoxygenation to predict (accurately) spheroid response's to re-oxygenation.

Response: We thank Reviewer 2 for this suggestion. We have not considered alternative more general functional forms to date, but agree that this is interesting future work and may allow for such predictions (R2.7a Page 18 Lines 406–411).

(R2.8) The results in Figure 5(k) are intriguing and seem to resemble cell blebbing. Have the authors looked at the modelling literature re cell blebbing? See, for example, TE Woolley et al, Biomech Model Mechanobiol (2014). Additionally, might a multiphase model which distinguishes necrotic, live and fluid phases be appropriate here (see, for example, work by TD Lewin)?

Response: We agree that these results are intriguing and are worth exploring in greater depth. In the discussion we now outline that interesting future work is to ascertain the physical and biological mechanisms that could explain these observations. In doing so we include references to Woolley et al. 2014 and works of Lewin et al. (Page 18 Lines 390–394).

(R2.9) Given that the experimental data distinguishes different phases of the cell cycle, the authors may be interested in considering how fluctuating oxygen environments affect cell cycle progress. See, for example, GL Celora et al, J Theor Biol (2022).

Response: We thank Reviewer 2 for sharing this interesting reference. We previously cited Celora et al. 2022 in the introduction. We now also reference Celora et al. 2022 in the discussion and state that interesting future work is to explore the impact of cyclic hypoxia on spheroid growth and cell cycle distribution within growing spheroids (Page 18 Lines 397–399).

In summary, I am happy to recommend the paper for publication, subject to minor revisions that address the minor comments and suggestions listed above.

Response: We thank Reviewer 2 for their recommendation that the paper be published subject to the minor revisions above.

Reviewer 3

Murphy et al use an extended Greenspan model for necrotic tumour development to investigate how temporal variation in conditions influences the development of a tumour. Specifically they add temporal variability to existing model parameters (for deoxygenation and re oxygenation) and then fit the model (under various hypotheses) to experimental data where the oxygenation of the tumour spheroid is varied over time.

The paper is well written and well presented. The topic is interesting to a general audience and, while I can't vouch for the experimental component, the modelling is sound and the parameter fitting is appropriate and well explained.

I would have liked to see more details on the parameter fits in the main paper but understand their position in the SI.

Response: We thank Dr James Osborne (Reviewer 3) for their positive summary of the paper.

There a few minor issues I have identified below.

(R3.1) Figure 2 I think it should be a) and d) which show snapshots

Response: We have corrected this typographical error in the caption of Figure 2.

(R3.2) Figure 2 What are the blue and orange lines in b and e is one a fit? Its not clear at present

Response: We now make clear in the caption of Figure 2 that Figures 2b and 2e are representative schematics of the oxygen partial pressure within a tumour spheroid under normoxic and hypoxic conditions, respectively. The solid blue/orange lines represent the oxygen partial pressure inside/outside of the hypoxic region and the hypoxic threshold is determined by the presence of pimonidazole staining (blue-dashed).
